# [Expression of Concern] Promoter methylation of death-associated protein kinase and its role in irradiation response in cervical cancer

**DOI:** 10.3892/or.2025.9028

**Published:** 2025-11-24

**Authors:** Rebecca Ching-Yu Leung, Stephanie Si Liu, Kelvin Yuen-Kwong Chan, Kar-Fai Tam, Kar-Loen Chan, Ling-Chui Wong, Hextan Yuen-Sheung Ngan

Oncol Rep 19: 1339–1345, 2008; DOI: 10.3892/or.19.5.1339

Following the publication of the above paper, it was drawn to the Editor's attention by a concerned reader that the blots showing β-actin mRNA expression in Fig. 1 on p. 1341 appeared to be similar to the β-actin mRNA blots shown in Fig. 3 on p. 1342, albeit with some horizontal and vertical resizing.

The authors were contacted by the Editorial Office to offer an explanation for this apparent anomaly in the presentation of the data in this paper; however, up to this time, no response from them has been forthcoming. Owing to the fact that the Editorial Office has been made aware of potential issues surrounding the scientific integrity of this paper, we are issuing an Expression of Concern to notify readers of this potential problem while the Editorial Office continues to investigate this matter further.

